# Audit of a retinopathy of prematurity screening programme in the Limpopo province, South Africa

**DOI:** 10.4102/jcmsa.v3i1.220

**Published:** 2025-08-15

**Authors:** Ntsakisi Bandi, Christopher Sutton, Tshilidzi van der Lecq

**Affiliations:** 1 Department of Surgery, Faculty of Health Sciences, University of Cape Town, Cape Town, South Africa; 2 Department of Paediatrics and Child Health, School of Medicine, University of Limpopo, Polokwane, South Africa

**Keywords:** retinopathy of prematurity, ROP screening, South Africa, sub-Saharan Africa, ROP prevalence

## Abstract

**Background:**

Retinopathy of prematurity (ROP) is a potentially blinding disorder. South Africa (SA) has national ROP screening guidelines to aid the timely diagnosis of infants requiring treatment. Several tertiary units in SA have published data on the prevalence of ROP; however, data from some provinces are lacking. An audit of ROP screening programmes, especially in these areas, is important to determine the ROP prevalence and whether screening is based on the national criteria.

**Methods:**

This retrospective audit included infants screened between 01 January 2019 and 30 June 2021 at a single tertiary unit in the Limpopo province, SA. The following criteria were used to identify eligible infants: birth weight (BW) < 1500 g or gestational age (GA) < 32 weeks.

**Results:**

A total of 203 infants were screened. The mean BW and GA were 1250 grams (s.d. 239.0) and 30.4 weeks (s.d. 2.43), respectively. Nine (4.4%) infants were diagnosed with ROP and 2 (1.0%) infants required treatment. Most (95.1%,193) infants met the screening criteria. Screening was completed in 158 (77.8%) infants and 44 (22.0%) were lost to follow-up (LTFU).

**Conclusion:**

Although eligible infants were identified based on the national criteria, a low prevalence of ROP was found among screened infants. This is likely because of lack of screening, late screening and high LTFU.

**Contribution:**

This study shows the value and importance of auditing ROP screening programmes even in countries with national screening guidelines to identify areas for improvement.

## Introduction

### Background

Retinopathy of prematurity (ROP) has been identified as a leading potentially preventable cause of childhood blindness across the world.^1^ Globally in 2010, an estimated 184 700 babies (of 14,9 million preterm babies) developed ROP, with 20 000 blind or severely visually impaired as a result.^2^ This prompted the World Health Organization’s (WHO) vision 2020 programme to identify ROP as one of the leading causes of avoidable childhood blindness.^3^ As a middle-income country, South Africa (SA) is experiencing the third epidemic of childhood blindness because of ROP, with a reported prevalence ranging from 13% – 33% among screened infants.^4,5,6,7,8,9,10^

Screening for ROP helps to detect those who need treatment. The CRYO-ROP study was the first study to promote the screening of infants by demonstrating that treatment could reduce unfavourable visual outcomes.^11^ Eyes with ‘threshold disease’ were randomised to either receive cryotherapy or observation. Unfavourable visual outcomes were found in 64.5% of control eyes compared to 44.7% of treated eyes (*p* < 0.001). In 2004, findings from the Early Treatment for ROP (ETROP) similarly demonstrated that treating eyes earlier (i.e., high-risk pre-threshold disease) with laser therapy or cryotherapy also reduced unfavourable visual outcomes (14.3% vs 19.8%, *p* < 0.005).^12^

In SA, the criteria used to identify those that require screening were published in the SA national guidelines in 2012.^13^ They specify that all premature infants meeting any of the following criteria require screening: a gestational (GA) < 32 completed weeks, or a birth weight (BW) < 1500 grams (g), or infants with a BW of 1500g–2000g with additional ROP related risk factors. In terms of the timing, screening should be performed at 4–6 weeks postnatal age (PNA) or at a postmenstrual age (PMA) of 31–33 weeks (whichever comes later). Lastly, they outline how screened infants should be followed up and managed. The introduction of these screening guidelines was shown to be beneficial in reducing the number of infants requiring treatment for ROP. Du Bruyn et al. published that the number of screened infants treated at a tertiary hospital in KwaZulu-Natal, changed from 8.75% in 2011 to 3.98% in 2012, and finally 2.36% in 2015. This study highlights the impact of implementing these guidelines in South Africa.^14^

Although there are well-developed ROP programmes throughout SA, it is important to audit programmes to monitor the prevalence of ROP, loss to follow up, and adherence to SA guidelines, especially because the prevalence of ROP can vary within a country.^14^ These data can enable comparison between centres and motivate for improvements in the ROP screening services.

Currently, there are no published data regarding the prevalence of ROP from any neonatal unit in the Limpopo province. The aim of the study was to determine the prevalence of ROP in infants born and screened for ROP at a tertiary unit in the Limpopo province and whether screening was conducted according to SA’s national ROP screening guidelines.

## Research methods and design

This study was a retrospective review of the ROP screening records of infants screened at a single tertiary unit located in Limpopo province, SA. Only infants born at the unit and screened between 01 January 2019 and 30 June 2021 were included in this study. Infants with incomplete medical records or those who were born at other facilities were excluded because of the absence of regular record-keeping for these patients.

In terms of the screening procedure, the screening programme aimed to adhere to the SA guideline recommendations. The examinations were conducted within the neonatal unit in a designated room well equipped with resuscitation equipment. The infants were dilated with cyclomydril drops and a topical anaesthetic agent was used as needed. Examinations were performed using binocular indirect ophthalmoscopy (BIO) by an ophthalmology medical officer supervised by a qualified ophthalmologist, on a weekly basis. Screening was considered to be completed based on the criteria in the national guidelines.

Examination findings were recorded on a standardised ROP screening form and stored in the medical records. Data about the infants booked for screening were obtained from the logbook. Demographic, neonatal and ophthalmic data collected from the screening records were recorded onto an Excel spreadsheet. The ophthalmic findings were classified according to ICROP^15^ and the decision to treat was based on the ETROP criteria.^12^

All data were analysed in JASP (0.17.1). Means, standard deviations and ranges were used to describe continuous variables, while frequencies and percentages were used to describe categorical variables.

### Ethical considerations

Ethical clearance to conduct this study was obtained, on 24 November 2021, from the University of Cape Town Human Research Ethics Committee (795/2021). A waiver of consent was approved for the analysis of the anonymised data. The study adhered to the principles of the Declaration of Helsinki (2008).

## Results

A total of 203 patients were screened for ROP and included in the study; one infant was excluded because of missing records. Table 1 presents the demographic characteristics for the entire cohort sample.

**TABLE 1:** Demographic characteristics of the entire cohort (*N* = 203).

Characteristic	*n*	%	Weeks	Grams	s.d.	IQR
**Gestational age at birth**						
Mean	-	-	30.4	-	2.43	-
Median	-	-	30.0	-	-	28.0–32.0
Range	-	-	24.0–38.0	-	-	-
**Birth weight**						
Mean	-	-	-	1250	239	-
Median	-	-	-	1260	-	1100–1400
Range	-	-	-	740–2100	-	-
**Gender**						
Male	95	46.8	-	-	-	-
Female	104	51.2	-	-	-	-
Undocumented	4	2.0	-	-	-	-
**Twin pregnancy**						
No	181	89.2	-	-	-	-
Yes	18	8.9	-	-	-	-
Other (Triplets)	4	2.0	-	-	-	-
**Stratified gestational age at birth (weeks)**						
≤ 24	2	1.0	-	-	-	-
25–27	20	9.8	-	-	-	-
28–30	84	41.4	-	-	-	-
31–33	80	39.4	-	-	-	-
34–36	15	7.4	-	-	-	-
Missing	2	1.0	-	-	-	-
**Stratified birth weight (grams)**						
≤ 1000	39	19.2	-	-	-	-
1001–1250	58	28.6	-	-	-	-
1251–1500	82	40.4	-	-	-	-
> 1500	23	11.3	-	-	-	-
Missing	1	0.5	-	-	-	-

s.d., standard deviation; IQR, interquartile range.

In this cohort, nine infants (4.4%) were diagnosed with an ROP. Those with ROP had a mean GA at birth of 29.5 weeks (s.d. 2.00, range 26–32). Their mean BW was 1202.2 grams (s.d. 230.8, range 800–1501). Only 2 (1.0%) infants developed Type 1 ROP and received treatment.

Some potential risk factors for ROP were recorded for the screened infants. These are displayed in Table 2.

**TABLE 2:** Risk factors noted among entire cohort (*N* = 203).

Category	*n*	%
**RDS**		
No	155	76.40
Yes	48	23.60
**Any sepsis**		
No	181	89.20
Yes	22	10.80
**Supplemental oxygen therapy**		
Yes	144	70.93
No	29	14.29
Absent	30	14.78

RDS, respiratory distress syndrome.

A total of 403 examinations were performed. The mean PNA at the time of the first screening examination was 6 weeks (s.d. 3.17, range 1–27). The mean PMA at the first screening examination was 36 weeks (s.d. 3.02, range 29–48). The first screening examination was performed on time (i.e., at a PNA ≤ 6weeks and/or a PMA ≤ 33 weeks) in 121 (59.6%) infants.

**FIGURE 1:**
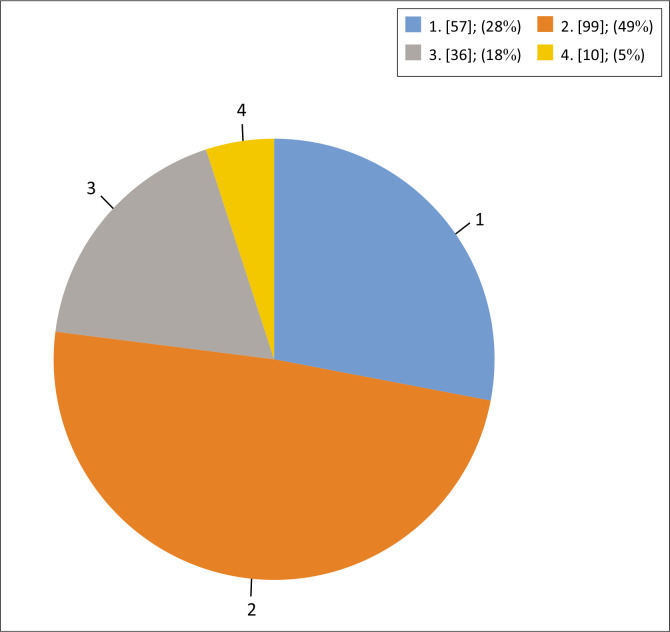
Number of screening examinations per infant (*N* = 202).

Most infants (99, 49%) received two screening examinations. Figure 1 shows the number of screening examinations received by each infant. One infant with an unclear number of examinations was excluded from the analysis.

**FIGURE 2:**
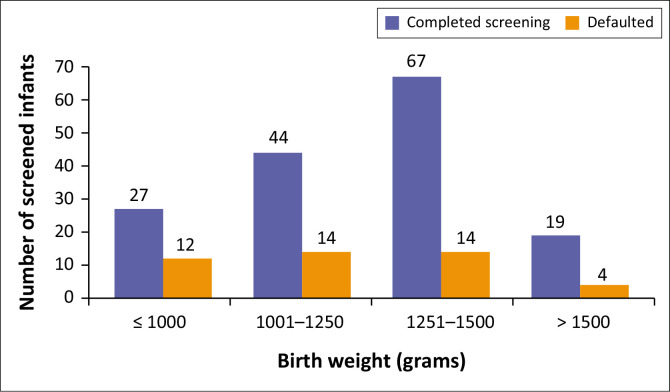
Birth weight stratification.

**FIGURE 3:**
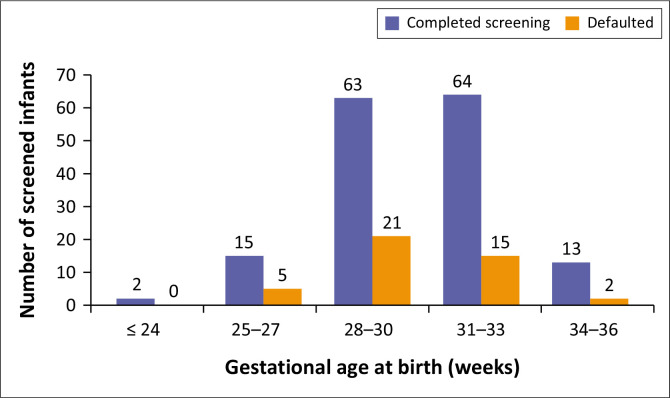
Gestational age stratification.

In terms of the screening outcome, 158 (77.8%) infants were fully screened and discharged. Figure 2 and Figure 3 show a stratification into the different BW and GA categories, while considering the outcome of the screening process. The proportion of infants who defaulted in BW category ≤ 1000g, 1001–1250g, 1251–1500g, > 1500g, were 30.8%, 24.1%, 17.3%, 17.4%, respectively. The proportions of infants who defaulted in GA categories ≤ 24 weeks, 25–27 weeks, 28–30 weeks, 31–33 weeks, 34–36 weeks were 0%, 25%, 25%, 19.0%, 13.3%, respectively.

## Discussion

This study aimed to audit the ROP screening programme and determine the prevalence of ROP in infants born and screened for ROP at a single tertiary unit located in Limpopo province, SA.

We found a 4% prevalence of ROP among the 203 screened neonates. This prevalence is considerably lower than that reported in other South African tertiary units. In the Western Cape, Keraan et al. diagnosed 40 cases (29.6%, *n* = 135) with ROP.^6^ Similarly, Visser Kift et al., in the 1104 screened infants, reported that 33.4% had ROP.^8^ In Gauteng, Mayet et al. diagnosed ROP in 84 cases (16.3%, *n* = 514).^9^ In a recent study performed in the same province, Seobi et al. compared the prevalence of ROP in infants born at the tertiary unit (53.1%, *n* = 1081) to those born and referred from regional units (46.9%, 954).^10^ The proportion of babies with ROP were 125 (11.6%) and 121 (12.7%), respectively. Lastly, Du Bruyn et al.’s study, based in KwaZulu-Natal, published a prevalence of 30.0% when analysing their cohort of 2734 infants over 8 years.^14^

In addition, our study also found a lower prevalence of treatment requiring ROP (2 infants, 1.0%) compared to the above studies. Keraan et al.’s study found that 2 (1.5%) infants required and received laser treatment.^6^ Visser Kift et al. reported that 27 (2.5%) infants required treatment.^8^ Mayet et al. indicated that 48.9% of infants who developed ROP were lost to follow-up (LTFU) and that 8 (1.6%) infants required treatment for ROP.^9^ On the other hand, Du Bruyn et al. reported the highest percentage of infants requiring treatment (4.94%, *n* = 135) over the 8 years of their study.^14^

Among the cohort of 203 neonates, 158 (77.8%) were fully screened and 44 (22%) were LTFU (i.e., defaulted). In the various birth weight categories screened, unfortunately, those with a BW ≤ 1000g had the highest proportion of LTFU (30.8%) when compared to the other categories. This group is known to be at the highest risk of requiring treatment for ROP.^11^ Only some SA studies have commented on the LTFU in their studies. Keraan et al.’s LTFU rate was 12.1% (*n* = 17), comparable to Mayet et al.’s 10.5% (*n* = 55).^6,9^ Visser Kift et al., Du Bruyn et al. and Seobi et al.^10^ did not comment on the LTFU rate.^8,10,14^

The low prevalence of ROP and treatment requiring ROP in our study is most likely because of reduced screening because of LTFU. The fact that infants referred from secondary-level facilities were excluded from our cohort may also contribute to the low prevalence. We also suspect that a significant proportion of infants requiring screening never presented for their first screening (email correspondence with the head of the neonatal unit). We estimate that during the study period, about 300 infants with BW ≤ 1000g and 900 very low birth weight infants (1000g – 1499g) were admitted in the unit.

A recent study reported findings on survival rates for preterm infants born in this unit.^16^ In this 2015 study, the survival rate was 30% for infants BW ≤ 1000g and 80% for the 1000g – 1499g group. Therefore, in the 3-year study period, about 90 infants (BW ≤ 1000g) and 720 in the 1000g – 1499g group leading to a total of 810 infants, should have been referred for screening. In our study, only 39 (43% of BW ≤ 1000g) and 140 (19% of 1000g – 1499g group) were screened at least once. There may be issues with identifying and referring eligible infants for screening as a result of absent referral protocols or a lack of awareness among neonatal staff. Also, for infants discharged before screening, poor parental education or lack of resources to attend the scheduled appointment are also possible reasons.

In terms of identifying infants eligible for screening, 193 (95.1%) infants met the screening criteria based on their GA or BW. Only 10 (4.9%) infants in the cohort had a GA > 32 weeks and a BW > 1500g. The infants received a total of 403 examinations. The mean PNA at the time of the first screening examination was 6 weeks (s.d. 3.17) with a range of 1–27 weeks. The mean PMA at the first screening examination was 36 weeks (s.d. 3.02) with a range of 29–48 weeks. In addition, the first examination was delayed according to the SA guidelines in about 40% of infants. This delay may have allowed ROP to resolve, also contributing to the low prevalence rate.

A limitation of the study was the exclusion of infants born outside the unit and referred to the unit for screening. This was a logistical issue as these infants are screened separately, not as part of the regular screening programme, making their ROP screening records difficult to obtain. Including these infants may have increased the prevalence of ROP. Other limitations include the retrospective nature of the study and the high LTFU.

## Conclusion

This study shows the value of an audit in providing information about the prevalence of ROP, the need for treatment, delays in screening and LTFU. This information can be monitored over time to help determine the effectiveness of an ROP screening programme, even those adhering to the SA national screening guidelines. In conclusion, this study found that in many ways the programme adhered to the SA guidelines, but detected a prevalence of ROP that falls under the range reported by similar units throughout the country. The low ROP prevalence suggested by this study serves as a starting point to determine the reasons for all the inefficiencies in the current programme, which can then be addressed and further monitored.
